# Immobilization of Allantoinase for the Development of an Optical Biosensor of Oxidative Stress States

**DOI:** 10.3390/s20010196

**Published:** 2019-12-29

**Authors:** Marialaura Marchetti, Luca Ronda, Riccardo Percudani, Stefano Bettati

**Affiliations:** 1 Centro Interdipartimentale Biopharmanet-TEC, Università di Parma, Parco Area delle Scienze 27/A, 43124 Parma, Italy; marialaura.marchetti@unipr.it (M.M.); stefano.bettati@unipr.it (S.B.); 2 Dipartimento di Medicina e Chirurgia, Università di Parma, Via Gramsci 14, 43126 Parma, Italy; 3 Dipartimento di Scienze Chimiche, della Vita e della Sostenibilità Ambientale, Università di Parma, Via Parco Area delle Scienze 11/A, 43124 Parma, Italy; riccardo.percudani@unipr.it

**Keywords:** silica gel, biosensor, allantoin, allantoinase

## Abstract

Allantoin, the natural end product of purine catabolism in mammals, is non-enzymatically produced from the scavenging of reactive oxygen species through the degradation of uric acid. Levels of allantoin in biological fluids are sensitively influenced by the presence of free radicals, making this molecule a candidate marker of acute oxidative stress in clinical analyses. With this aim, we exploited allantoinase—the enzyme responsible for allantoin hydrolization in plants and lower organisms—for the development of a biosensor exploiting a fast enzymatic-chemical assay for allantoin quantification. Recombinant allantoinase was entrapped in a wet nanoporous silica gel matrix and its structural properties, function, and stability were characterized through fluorescence spectroscopy and circular dichroism measurements, and compared to the soluble enzyme. Physical immobilization in silica gel minimally influences the structure and the catalytic efficiency of entrapped allantoinase, which can be reused several times and stored for several months with good activity retention. These results, together with the relative ease of the sol-gel preparation and handling, make the encapsulated allantoinase a good candidate for the development of an allantoin biosensor.

## 1. Introduction

Oxidative stress is defined as an imbalance between the presence of reactive species and the antioxidant body response. Recently, much evidence proved the correlation of oxidative stress with several pathological conditions (cardiovascular diseases, diabetes, arthritis, neonatal hypoxia, etc.) [1,2,3,4,5], highlighting the necessity to find specific biomarkers for its evaluation. This increasing exigency combines with the preferential use of non-invasive methods for sample collection, electing saliva and urine as best alternatives in clinical biochemistry. In this context, the attention is progressively focusing on allantoin, a stable and polar small molecule generated in humans from the non-enzymatic oxidation of uric acid, as a potential biomarker for routine clinical analyses. Indeed, uric acid is the end product of purine metabolism in humans and one of the main constituents of the total antioxidant capacity (TAC) in body fluids [6,7,8,9], representing the most active and abundant scavenger of hydrophilic reactive oxygen and nitrogen species and contributing to 35–65% of the plasma TAC [10,11,12]. Since allantoin spontaneously forms upon non-enzymatic urate degradation, allantoin levels increase promptly responding to oxidative stress conditions and can be measured in different biological fluids, e.g., plasma, synovial fluid, saliva and urine [5,13,14]. The small size and the high polarity of allantoin, however, make its quantification challenging, thus limiting its exploitation as a biomarker. The general methods used for allantoin measurements require sensitive and specific instrumentations and exploit critical or time-consuming techniques like chemical derivatization, gas or liquid chromatography associated with mass spectrometry (GC/LC-MS), capillary electrophoresis and enzyme cycling assays [3,4,8,15,16,17,18,19,20,21]. To overcome these limits, we proposed a novel assay based on the enzymatic production of allantoate from allantoin, and its subsequent chemical conversion into a fluorescent compound, detectable with a bench fluorescence plate reader [22]. The enzymatic step is performed by the recombinant enzyme allantoinase from *Pseudomonas fluorescens* (puuE, E.C. 3.5.2.5) [23], able to stereoselectively convert (S)-allantoin into allantoate, without any influence of the reverse reaction. The assay specificity is granted by (i) the known reaction specificity of the enzyme, and (ii) the removal of the contribution of interferent molecules through the subtraction of the baseline, which is generated by performing the same chemical step of the complete assay, while skipping the enzymatic reaction, so that the detected signal is only due to the allantoate generated from allantoinase reaction with allantoin. In this work, we propose the immobilization of puuE to explore the possibility to develop a reusable biosensor. The relevance of a biosensor resides in the selectivity and sensitivity in target quantification and reusability, determining its applicability as a tool for routine clinical practice. With this purpose, we entrapped puuE in a wet nanoporous silica gel matrix, a strategy largely exploited in enzyme immobilization because of its several advantages, among which the easy and flexible chemistry, with mild chemical and physical entrapment conditions compatible with protein stability, the optical transparency allowing signal detection with conventional spectroscopic techniques, the ease of separation of the active material from the reaction solution [24,25,26,27,28,29,30,31].

During the reaction of polymerization, the protein molecules enclosed in the sol-gel drive the shaping of the matrix pores. The resulting nanoporous structure prevents protein leaking while allowing diffusion of reactants and products but—together with altered solvent microviscosity within the pores—poses steric and thermodynamic constraints in the conformational equilibrium of the enzyme. While enzyme encapsulation in silica gels does not affect substrate specificity, it can potentially result in the immobilization of poorly or differently active and stable conformations [32,33,34,35,36,37,38,39,40]. We therefore assayed allantoinase structural features and stability after encapsulation and the influence of the silica gel matrix on enzymatic activity, in order to verify the approach suitability for the setup of a biosensor. The presented results demonstrate the applicability of this strategy to a chemical/enzymatic assay for allantoin quantification [22], in which respect it represents a major improvement in terms of reusability and storability.

## 2. Materials and Methods

### 2.1. Enzyme Expression and Purification

Recombinant puuE from *P. fluorescens* was expressed in *Escherichia coli* host, as previously described [23]. Cells were lysed in 50 mM sodium phosphate, 300 mM NaCl, 10% glycerol (v/v), 200 µM phenylmethylsulfonyl fluoride, 200 µM benzamidine and 1.5 µM pepstatin A, pH 7.6, and sonicated by 5-s bursts alternated to 1-min pauses. Surnatant was filtered with 0.2 µM filter units and concentrated by Amicon Ultra-15 centrifugal filter devices (Merck-Millipore, Darmstadt, Germany) with 30 kDa-cutoff. puuE was purified to homogeneity by gel filtration chromatography on a BioSepra Ultrogel AcA 44 column (PALL Life Sciences, Port Washington, NY, USA) in 100 mM potassium phosphate, 150 mM NaCl, pH 7.6. Fractions containing the enzyme were aliquoted, flash-frozen in liquid nitrogen and stored at −80 °C until further use.

### 2.2. Enzymatic Activity

The hydrolase activity was followed recording single-wavelength kinetics at 210 nm at 37 °C with a J715 spectropolarimeter (Jasco, Tokyo, Japan) equipped with a Peltier thermostatic cell. Reactions were carried out in a 1 mm pathlength quartz cuvette, in 20 mM potassium phosphate, pH 7.4, in the presence of 70 nM enzyme (monomer concentration). The experimental buffer is optimized to avoid instrumental interferences.

### 2.3. Enzyme Encapsulation

puuE was encapsulated in a silica matrix by the sol-gel method [24,25]. Tetramethylorthosilicate (TMOS), water and a 40 mM HCl solution were mixed and sonicated for 20 min (divided in 5-min on and 1-min off cycles), then an equal volume of 10 mM phosphate, pH 6.0, was added and the solution was fluxed with humidified nitrogen for 40 min. The obtained sol was mixed in a 1:1 ratio with puuE in 100 mM phosphate buffer, 150 mM NaCl, pH 7.6, and layered either on the bottom of 2 mL glass vials, or on the non-optic wall of quartz cuvettes, or on quartz slides. The increase of pH promotes polymerization and consequent solidification of the mixture, which occurred in about two minutes at 4 °C. Upon polymerization, vials were maintained in 100 mM phosphate, 150 mM NaCl, pH 7.0 at 4 °C and used after at least 24 h of silica matrix aging.

### 2.4. Secondary and Tertiary Structure Measurements

Circular dichroism (CD) spectra were collected with a Jasco J715 spectropolarimeter between 200 and 260 nm in a 1 cm pathlength quartz cuvette, thermostated at 20 °C, in 20 mM potassium phosphate, pH 7.4. Measurements of puuE in solution were recorded at 0.35 µM monomer concentration. Spectra on puuE in silica gel were collected by encapsulating 29 µg of recombinant enzyme on the 2 cm^2^-surface of a quartz slide, adhered to one of the optic faces of the cuvette. Fluorescence emission spectra upon excitation of tryptophan residues at 298 nm were collected by a Fluoromax-3 fluorometer (HORIBA Jobin Yvon, Kyoto, Japan) between 310 nm and 550 nm at 20 °C, with slits set to optimize the signal-to-noise ratio. Measurements of puuE in solution were recorded at 0.2 µM monomer concentration, in 20 mM potassium phosphate, pH 7.4. Front-face emission spectra were recorded on puuE encapsulated onto the quartz slides (vide supra) in a 1 cm pathlength quartz cuvette.

### 2.5. Stability Measurements

puuE stability after encapsulation was assessed both over time and after several cycles of reuse of the protein-doped silica gel. Before use, the silica matrix was rinsed once with storage buffer, crumbled, and washed with the same buffer. After washing, the buffer was discarded and 0.75 mL of 0.2 mM racemic allantoin in 20 mM potassium phosphate, pH 7.4 were added to the encapsulated enzyme (8 µg, 0.3 µM final monomer concentration). The solution was then incubated under agitation for 10 min on a thermostated Peltier plate set at 37 °C. For the reuse, after each incubation the gel was rinsed twice with 0.75 mL of storage buffer. The enzymatic activity was checked collecting CD spectra between 200 nm and 340 nm on the final solution, at 20 °C in a 2 mm quartz cuvette. The experiment was carried out in triplicate for each time point. For comparison, a solution of 23.4 µM puuE was stored over time in storage buffer, at 4 °C. The enzymatic activity was tested at 0.3 µM (final monomer concentration) in reaction buffer, in 2 mL glass vials. After 10 min under agitation at 37 °C, reaction solutions were stopped trough diafiltration in Amicon Ultra 0.5 mL ultracentrifuge devices with 30 kDa-cutoff (Merck-Millipore, Darmstadt, Germany). CD spectra were recorded on the flow-through solution.

### 2.6. Fluorescent Quantification of Allantoin by Immobilized Allantoinase

A calibration curve was built adding increasing concentrations of racemic allantoin to 100 mM potassium phosphate buffer, pH 7.4 (final range 0.5–50 µM). A reaction in the absence of allantoin was carried out for blank subtraction. The assay was performed as previously described [22]. Briefly, recombinant puuE was encapsulated on the internal part of PCR strip caps following the protocol previously described in this section, using 300 ng of enzyme for each cap (final volume 2 µL). Upon polymerization, caps were maintained overnight in storage buffer at 4 °C. 30 µL of solutions containing increasing concentrations of allantoin were added into 0.2 mL PRC tubes, closed with the allantoinase functionalized caps, and put upside down at 37 °C for 15 min [22]. After the enzymatic conversion of allantoin to allantoate, caps were substituted with normal ones, 120 µL of 6 M HCl and 3.5 µL of 5% resorcinol (w/v) were added and the samples were incubated in a thermocycler at 100 °C for five minutes. After cooling down the reactions in ice for two minutes, 2.5 µL of each sample were added to 239 µL of 100 mM diethanolamine, pH 9.6, on a polystyrene 96-well black microplate (Greiner BioOne, Kremsmünster, Austria), after the addition of 9 µL of a 1.5 M ascorbate solution. The multiwell plate was read by a Spark 10M (Tecan, Männedorf, Switzerland) plate reader recording signal emission at 535 nm upon excitation at 490 nm to detect the formation of the fluorescent compound 2,2′,4,4′-tetrahydroxy-diphenylacetic acid, with an absorption peak centered at 490 nm and an emission peak centered at 530 nm. Reactions were carried out in triplicate and the revelation on the plate was made in duplicate.

## 3. Results and Discussion

### 3.1. Effect of Silica Gel Encapsulation on Allantoinase Secondary and Tertiary Structure

To determine whether the encapsulation process affected puuE structure and activity, we characterized the spectroscopic and functional properties of enzyme after entrapment in comparison to the free allantoinase. To this goal, we exploited far-UV circular dichroism spectra and tryptophan fluorescence emission, which are sensitive probes of protein secondary and tertiary structure, respectively.

**Figure 1:**
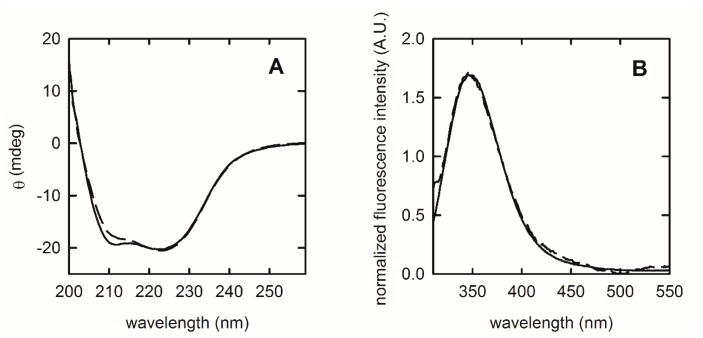
Structural characterization of entrapped puuE (solid lines) in comparison to the free enzyme (dashed lines) in 20 mM potassium phosphate, pH 7.4. (**A**) Normalized far-UV circular dichroism spectra; (**B**) normalized tryptophan emission spectra upon excitation at 298 nm.

As determined by structural studies, native puuE is a homotetramer and each monomer is folded in a deformed (β/α) barrel structure, generating an independent active site [23]. The α-helices constitute around 40% of the structure, whereas around 10% of the sequence folds as β-strands. Recombinant allantoinase was mixed in a 1:1 ratio with the sol (see Section 2.3) and, before polymerization, deposited as a thin layer on a quartz slide. The resulting puuE-doped gel was used for the structural characterization by circular dichroism (CD) and static fluorescence spectroscopy (Figure 1), after at least 24 h of aging.

The CD spectrum of free allantoinase (Figure 1A, dashed line) presents a strong positive band at wavelengths below 200 nm and two negative peaks centered at 211 and 223 nm, typical of an α/β folding, in accordance with the crystallographic data. For comparison, the embedded enzyme (Figure 1A, solid line) does not reveal significant alterations in the secondary structure, with only minor differences in the region between 205–215 nm that could be attributed to very slight changes in helical content or scattering effects due to the gel matrix.

**Figure 2:**
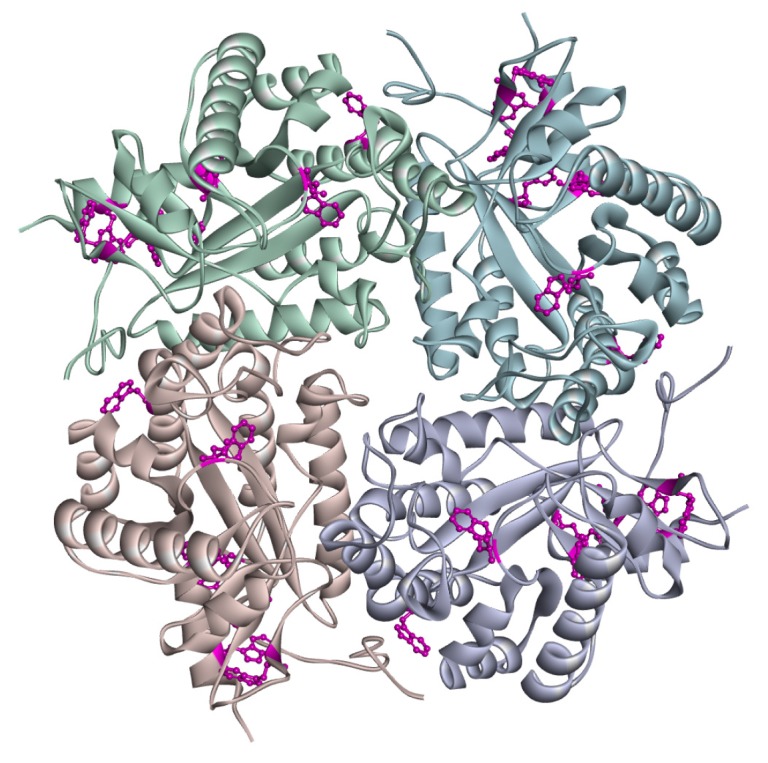
Representation of puuE tetrameric assembly (Protein Data Bank 3cl6). Monomers are shown as solid ribbons in different colors, while tryptophan residues are evidenced in purple ball and stick.

Fluorescence emission of tryptophan residues is sensitive to the microenvironment and, therefore, protein structural changes can be monitored as shifts in their maximum emission wavelength exploiting their solvatochromic nature. In general, the selective excitation of this amino acid in proteins provides an emission maximum ranging from about 310 nm to about 355 nm, based on solvent polarity and the level of solvent exposure [41,42]. Twenty-eight tryptophan residues are present in a biological unit of puuE (Figure 2). To assess the effect of encapsulation on puuE tertiary structure, we selectively excited at 298 nm the embedded allantoinase on a quartz slide (Figure 1B, solid line) and, for comparison, the enzyme in solution (Figure 1B, dashed line). The two spectra are completely superimposable, with the maximum emission peak centered at 348 nm, indicating an overall conservation of the native folding.

### 3.2. Analysis of Catalytic Parameters and Influence of Silica Gel Matrix on puuE Activity

The activity of an enzyme is defined through the determination of two main parameters: the turnover number (k_cat_), representing the number of substrate molecules processed in a time unit, and the Michaelis constant (K_M_), roughly representing the enzyme apparent affinity for its substrate. From these two values it is possible to obtain the catalytic efficiency (also known as specificity constant, k_cat_/K_M_), which is best used as comparative parameter between enzymatic activities in different conditions [34,43,44,45]. More specifically, catalytic efficiency represents the efficiency of the encounter complex to form and give the reaction product. This value for allantoinase is around 7.5 × 10^4^ M^−1^·s^−1^ and falls in the median range of reported enzymatic activities belonging to the primary metabolism of nucleotides [46].

**Figure 3:**
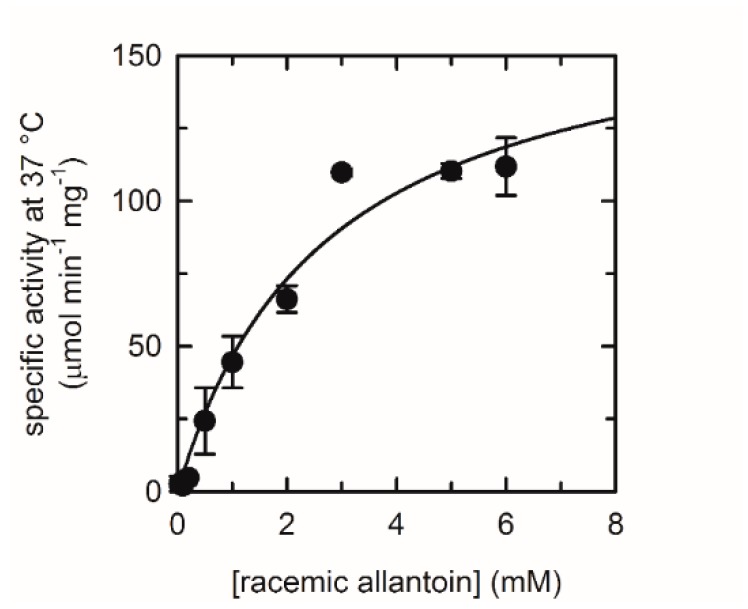
Michaelis–Menten dependence of puuE activity in the presence of increasing concentrations of racemic allantoin. Single-wavelength kinetics of (S)-allantoin consumption were recorded at 210 nm in 20 mM potassium phosphate, pH 7.4, at 37 °C. Each point corresponds to a separate (S)-allantoin consumption kinetics; error bars represent standard errors of experimental replicates.

The catalytic parameters of the free puuE were calculated by fitting the enzymatic initial rates in the presence of increasing allantoin concentrations to the Michaelis–Menten dependence equation (Figure 3, Table 1). CD kinetics were collected following the decreasing signal at 210 nm, corresponding to (S)-allantoin consumption. Racemization rate of the residual (R)-allantoin was considered negligible in the time windows required for the kinetics measurements [47,48]. Moreover, the reverse stereoselective condensation of allantoate to form (S)-allantoin does not affect the kinetic signal, because of the lesser puuE efficiency in catalyzing this reverse reaction [23]. Conversely, the direct measurement of encapsulated puuE activity was limited by allantoin molar ellipticity coefficient and the experimental setup. In fact, measurements were carried out encapsulating puuE onto the non-optical face of a 1 cm pathlength quartz cuvette, and the solution transmittance limited to 40 µM racemic allantoin the maximum concentration feasible in these assay conditions. For these reasons, the catalytic efficiency of allantoinase entrapped in the silica gel matrix was obtained indirectly by the application of the equation:

[S] = [S_0_]e^−kt^ (1)

With

K = (k_cat_/K_M_)[E] (2)

to 4.37 µM puuE in 50 µL of sol-gel matrix, in the presence of 750 µL of 40 µM racemic allantoin. This procedure is applicable when the concentration of substrate is sensitively lower than the K_M_ value [49]. The accuracy of this indirect determination was tested extrapolating the catalytic efficiency of the free enzyme by Equation (1) to the lower substrate concentrations of the Michaelis–Menten dependence. The k_cat_/K_M_ value calculated in this way was in good agreement with the one obtained from the ratio of the experimentally calculated parameters.

The apparent catalytic efficiency of the embedded allantoinase is lower than that calculated for the free enzyme, but within the same order of magnitude (Table 1). This difference can be ascribed to steric effects consequent to the immobilization, limiting functionally relevant enzyme dynamics/conformational equilibria, or to an excessive sol-gel thickness making catalysis at least partially rate-limited by substrate diffusion.

Therefore, although the overall efficiency of the encapsulated enzyme is satisfying and makes puuE suitable for biosensing applications, it is significantly different from the free enzyme. Hence, we wanted to further investigate the impact of sol-gel matrix thickness on diffusional limit and, consequently, on allantoinase activity. With this aim, we immobilized equal amounts of puuE layering different volumes on one of the non-optical faces of a 1 × 1 cm quartz cuvette. The thickness of the layers was calculated considering a 3.3 cm^2^ coated area and the different sol-gel volumes employed, assuming a parallelepiped as a shape.

Matrix diffusion limit can be determined from the catalytic parameters of the enzyme and the concentration of puuE in the sol-gel, applying the following equation [34,50]:

d_c_ = {[(K_M_ + [S_0_]) × D’]/(k_cat_ × [E])}^1/2^ (3)

where d_c_ is the matrix critical thickness over which rates are diffusion-controlled, K_M_ and k_cat_ are the catalytic parameters of puuE in solution (Table 1), [E] is the enzyme concentration and [S_0_] the substrate concentration. D′ is the diffusion coefficient of the substrate inside the gel calculated as:

D’/D = 1 − (a^2^/r) (4)

where a is the average molecular radius (5.42 Å for allantoin), D (6 × 10^−6^ cm^2^/s) is the diffusion coefficient in water for compounds with a molecular weight around 200 Da, and r (40–50 Å) is the average pore radius of the gel.

**Figure 4:**
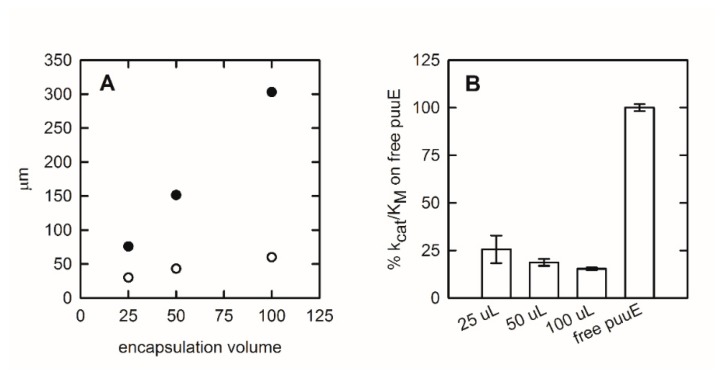
Silica matrix parameters and puuE activity after entrapment in different sol-gel volumes. (**A**) Comparison between sol-gel thickness (closed circles) and matrix critical thickness (open circles) immobilizing 7.75 µg of puuE in increasing matrix volumes. (**B**) Catalytic efficiency of puuE encapsulated in increasing matrix volumes with respect to the free enzyme. For each condition of encapsulation, the experiments were carried out in triplicate and data were collected from single-wavelength CD kinetics at 210 nm in the presence of 0.2 mM racemic allantoin 20 in mM potassium phosphate, pH 7.4, at 37 °C.

As can be noticed from values reported in Table 2 and in Figure 4A, the matrix critical thicknesses calculated for the different encapsulation volumes (open circles) are systematically lower than the real dimensions of the layers (closed circles). As expected, the catalytic efficiency is inversely proportional to the immobilization volume and, consequently, thickness. The 25% catalytic efficiency (with respect to the soluble enzyme) of the 25 µL enzyme represents a lower limit for the encapsulated enzyme, indicating that the effect of entrapment and matrix physical properties on enzyme function is not dramatic.

### 3.3. Application of the Encapsulated Enzyme as a Biosensor

**Figure 5:**
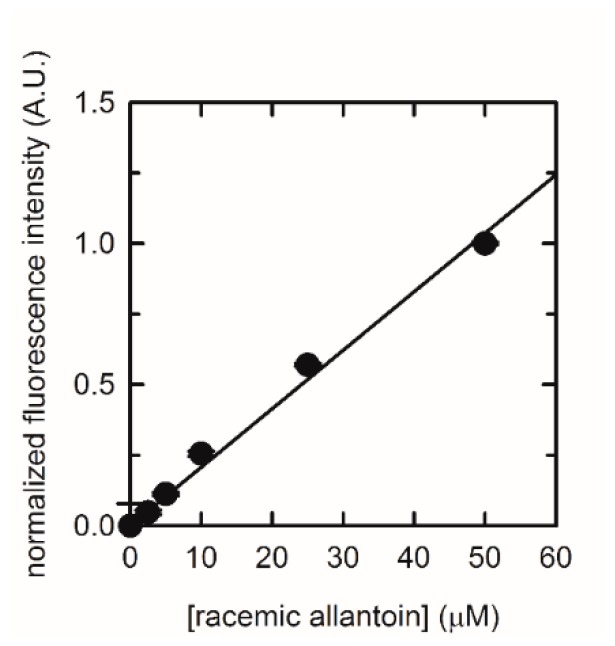
Normalized fluorescence emission at 530 nm (λ_ex_ = 490 nm) detected by microplate reader at increasing concentrations of allantoin in 100 mM phosphate buffer, pH 7.4, enzymatically processed by encapsulated puuE. Linear regression gave a R^2^ of 0.99.

The effective suitability of immobilized puuE in biosensing applications was tested through its application in the enzymatic step of an allantoin quantification assay recently developed by our group [22]. Thin layers of silica gel containing 300 ng of recombinant puuE were layered on the internal part of the caps of 0.2 mL vials. Increasing amounts of racemic allantoin, ranging from 0.5 to 50 µM in 100 mM potassium phosphate, pH 7.4, were converted into allantoate by incubating samples upside down in 0.2 mL vials sealed by the functionalized caps. After a fixed reaction time, caps were removed and stored for reuse [22]. The fluorescence values obtained from the plate reading were subtracted of the blank (a reaction performed in buffer in the absence of allantoin) and normalized, and their dependence on allantoin concentration was fitted to a linear equation (Figure 5). The analysis provided a linear response in the explored range, confirming that encapsulated allantoinase can represent a valuable option in the proposed routine assay for allantoin quantification.

### 3.4. Reusability of the Biosensor and Stability over Time

**Figure 6:**
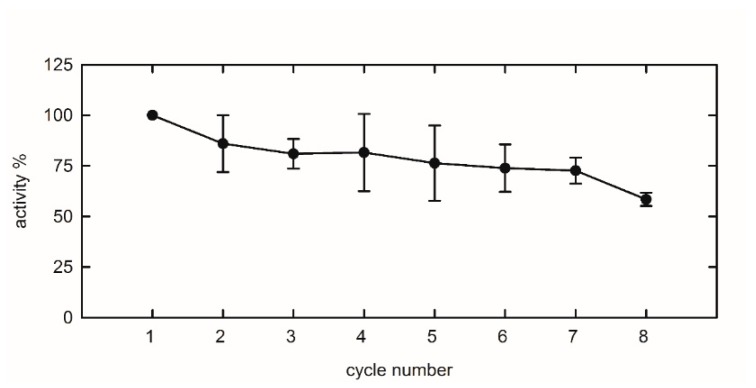
Repeated uses of immobilized puuE. Reactions were carried out at 37 °C in 20 mM potassium phosphate, pH 7.4, in the presence of 0.2 mM racemic allantoin, and stopped after a fixed time of 10 min. Reported percentage values refer to (S)-allantoin consumption as monitored at 210 nm through CD spectra. The mean value of three reactions at cycle 1 represents the reference for the normalization of subsequent cycles. Data are represented as mean ± s.d. and lines are for eye-guidance only.

Generally, one of the pursued advantages in using an immobilized enzyme is its increased stability and, as a consequence, its disposition to long-term storage and reusability. We tested this feature in our allantoin biosensor, employing the same encapsulated puuE batch for several cycles of reuse. The reactions were performed on aliquots of the same batch containing 0.2 mM racemic allantoin in 20 mM potassium phosphate, pH 7.4, and the consumption of (S)-allantoin was checked by recording the CD signal of the solutions. The end-point values after ten minutes of reaction were converted into activity percentage with respect to the first cycle (Figure 6). The reported graph shows how, after eight cycles of reuse, allantoinase retains more than 50% of the initial activity, indicating that this strategy is applicable to the development of a recyclable enzymatic device for allantoin quantification.

**Figure 7:**
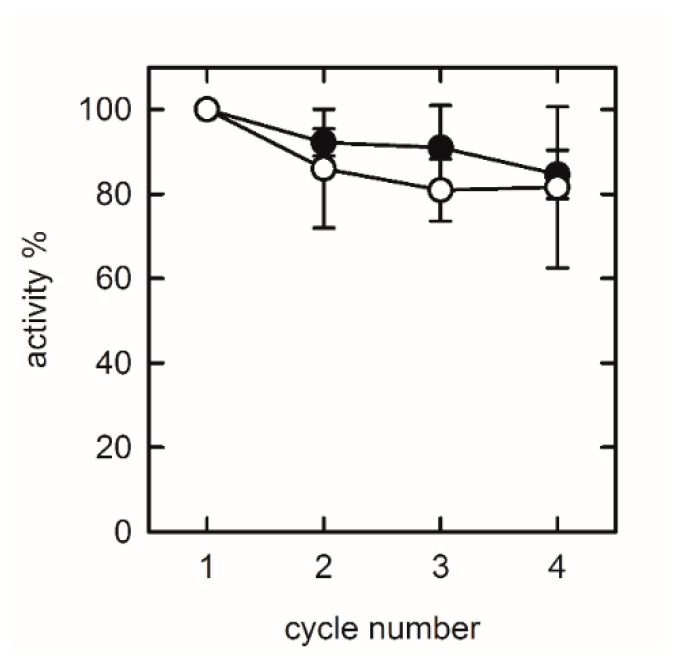
Effect of NaCl on biosensor reuse. Before each cycle, the puuE-doped gel was rinsed in 100 mM potassium phosphate buffer, pH 7.0, in the presence (open circles) or in the absence (closed circles) of 150 mM NaCl. Data were collected from single-wavelength CD kinetics at 210 nm in the presence of 0.2 mM racemic allantoin in 20 mM potassium phosphate, pH 7.4, at 37 °C. Activity is expressed as percentage with respect to the first cycle of use. Data are represented as mean ± s.d. of three replicates and lines are for eye-guidance only.

During the setting of the experimental conditions, we found that the presence of NaCl in the sol-gel storage and washing solution is not essential for allantoinase stability. In fact, as shown in Figure 7, after four cycles of reuse the percentage of activity is almost the same either if the puuE-doped silica gel is rinsed with a potassium phosphate buffer containing 150 mM NaCl or if the salt is absent. As previously observed for other encapsulated proteins [51,52,53,54], the role of counter-ions in shielding the excess negative charges exposed on the surface of gel pores at near physiological pH is important to avoid partitioning of polar solutes and aspecific protein-pore interactions, a possible source of functional heterogeneity for entrapped molecules. In this case, electrostatic interactions between the pores and charged groups on protein surface seem not to limit functionally relevant dynamics.

**Figure 8:**
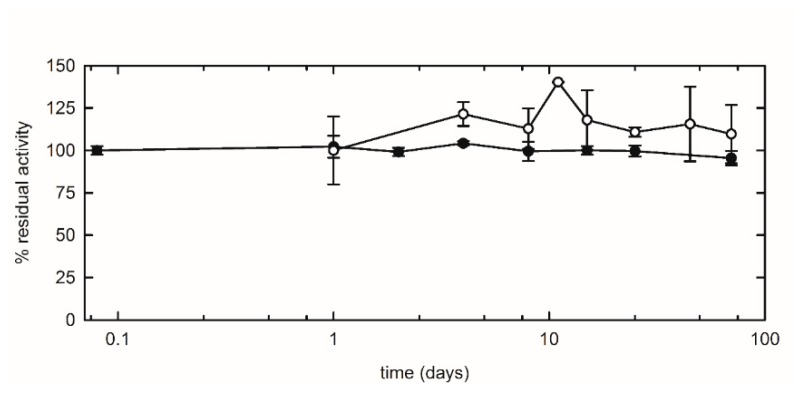
Comparison between free (closed circles) and encapsulated (open circles) puuE catalytic activity over storage time. Reactions were carried out at 37 °C in 20 mM potassium phosphate, pH 7.4, in the presence of 0.2 mM racemic allantoin, and stopped after a fixed time of 10 min. The mean value of three reactions at day 0 (free puuE) and day 1 (encapsulated puuE) represent the reference for the normalization of subsequent time points. The activity of immobilized puuE was recorded from day 1 because of the time required for silica gel matrix aging, and for each time point a new aliquot of puuE encapsulated on day 0 was used. Data are represented as mean ± s.d. of three replicates and lines are for eye-guidance only.

The long-term storage stability of encapsulated puuE was assessed as an additional central condition for its application as a biosensor. The enzymatic activity of single-use batches of entrapped allantoinase was monitored for several months and the results were compared with the free enzyme stored in solution at 4 °C. Allantoinase demonstrated to be a remarkably stable enzyme per se, and its stability was retained even after encapsulation, keeping an almost unaltered activity over three months of storage (Figure 8).

## 4. Conclusions

Allantoin is a promising biomarker for the evaluation of oxidative stress states. Currently available analyses for its quantification are time-consuming and mostly performed by GC/LC-MS techniques, requiring specific and expensive instrumentation. In this context, we developed a fast enzymatic-chemical assay aimed at simplifying allantoin quantification by fluorescence revelation. To further improve our assay, we decided to immobilize allantoinase, the enzyme responsible for the initial allantoin processing, conceiving a reusable biosensor. puuE from *P. fluorescens* was successfully entrapped in a nanoporous silica gel matrix, preserving its secondary and tertiary structure. The catalytic efficiency was also conserved, with gel thickness not dramatically affecting enzymatic activity with respect to the allantoinase free in solution. Our data show puuE to be a highly stable enzyme, retaining an almost unvaried activity for several months. This feature is preserved after encapsulation. The entrapment in a solid matrix allows the reusability of allantoinase for several reactions, with the retention of more than 50% of activity after eight cycles of reuse. These results support the development of an allantoinase-based biosensor for allantoin quantification in biological samples.

## Figures and Tables

**Table 1:** Catalytic parameters and catalytic efficiency of free and encapsulated puuE on (S)-allantoin in 20 mM potassium phosphate, pH 7.4, at 37 °C. For the free enzyme, k_cat_ and K_M_ values were obtained by fitting kinetic data to the Michaelis–Menten equation and the catalytic efficiency was calculated as the ratio between the turnover number and Michaelis constant. Catalytic efficiency for entrapped puuE was obtained on single kinetics in the presence of 40 µM racemic allantoin, applying Equation (1).

puuE	k_cat_ (s^−1^)	K_M_ (mM)	k_cat_/K_M_ (M^−1^ s^−1^)
Free	101.57 ± 14.26	1.35 ± 0.43	7.52 ± 2.62 × 10^4^
Silica gel	—	—	1.27 ± 0.12 × 10^4^

**Table 2:** Thickness and matrix critical thickness (d_c_) of different sol-gel volumes containing 7.75 µg of puuE. Matrix thickness was calculated from encapsulation volume assuming 3.3 cm^2^ as the base area of a parallelepiped; d_c_ values were calculated applying Equations (3) and (4) and using catalytic parameters of free puuE listed in Table 1.

Volume (µL)	[puuE] in Gel (µM)	Thickness (µM)	d_c_ (µM)	k_cat_/K_M_ (M^−1^ s^−1^)
25	8.75	75.8	30	1.74 ± 0.49 × 10^4^
50	4.37	151.5	43	1.27 ± 0.12 × 10^4^
100	2.19	303.0	60	1.04 ± 0.04 × 10^4^
